# Next generation sequencing shows high variation of the intestinal microbial species composition in Atlantic cod caught at a single location

**DOI:** 10.1186/1471-2180-13-248

**Published:** 2013-11-09

**Authors:** Bastiaan Star, Thomas HA Haverkamp, Sissel Jentoft, Kjetill S Jakobsen

**Affiliations:** 1 Centre for Ecological and Evolutionary Synthesis (CEES), Department of Biosciences, University of Oslo, PO Box 1066, Blindern, N-0316 Oslo, Norway

## Abstract

**Background:**

The observation that specific members of the microbial intestinal community can be shared among vertebrate hosts has promoted the concept of a core microbiota whose composition is determined by host-specific selection. Most studies investigating this concept in individual hosts have focused on mammals, yet the diversity of fish lineages provides unique comparative opportunities from an evolutionary, immunological and environmental perspective. Here we describe microbial intestinal communities of eleven individual Atlantic cod (*Gadus morhua*) caught at a single location based on an extensively 454 sequenced 16S rRNA library of the V3 region.

**Results:**

We obtained a total of 280447 sequences and identify 573 Operational Taxonomic Units (OTUs) at 97% sequence similarity level, ranging from 40 to 228 OTUs per individual. We find that ten OTUs are shared, though the number of reads of these OTUs is highly variable. This variation is further illustrated by community diversity estimates that fluctuate several orders of magnitude among specimens. The shared OTUs belong to the orders of *Vibrionales,* which quantitatively dominate the Atlantic cod intestinal microbiota, followed by variable numbers of *Bacteroidales*, *Erysipelotrichales, Clostridiales, Alteromonadales* and *Deferribacterales*.

**Conclusions:**

The microbial intestinal community composition varies significantly in individual Atlantic cod specimens caught at a single location. This high variation among specimens suggests that a complex combination of factors influence the species distribution of these intestinal communities.

## Background

The intestinal microbial community provides a variety of crucial functions for their vertebrate hosts e.g. [1], though the factors that influence the colonization of this habitat are less understood. Common patterns among microbial communities of different hosts have promoted the concept of a core set of species, which provides a minimal functionality in the healthy gut and which is determined by host-specific selection [2,3]. For example, host transcriptional responses to microbial colonization appear to be conserved among a wide range of vertebrates, including fish [4]. Moreover, within the intestinal community of humans, some species are more prevalent [3,5,6] and functional gene profiles are highly similar among individuals [7]. Nevertheless, the utility of the core microbiota concept at a fine taxonomic level has recently been questioned due to limited evidence of universally abundant species in humans [8,9].

Fish provide unique opportunities to investigate the factors that influence the composition of the vertebrate intestinal microbiota due to their high species diversity [10], dietary variation or habitat preferences [11], and divergent immune architecture. For instance, considering the differences in immune systems as an example, Atlantic cod lacks the antigen presenting major histocompatibility complex (MHC) II system, which was thought to be conserved among all jawed vertebrates [12]. This lack of MHC II may affect the interactions of Atlantic cod with its microbial community [13]. A extensive meta-analysis -based on uncultured and cultured sampling methods- indicates that the composition of the intestinal communities in teleosts is influenced by both abiotic and biotic factors [11]. Nevertheless, this meta-analysis is predominantly based on pooled Sanger sequencing data, and studies investigating microbial communities in fish using high-throughput sequencing are relatively rare. Moreover, the studies that employed these methods so far have focused on fresh water species held in semi-controlled environments [14-16]. One exception investigating natural populations of zebrafish, identified a core intestinal microbiota based on shared Operational Taxonomic Units (OTUs), despite substantial differences in host provenance and domestication status [17]. This study pooled 4, 6 and 20 individuals respectively, before sequencing [17]. Therefore, to our knowledge, a characterization of the microbial community using high-through methodologies in wild-caught, individual fish is still lacking. Here we investigate the intestinal microbial communities of 11 wild-caught Atlantic cod collected at a single location and quantify a core microbiota based on shared membership in a 454 sequenced 16S rRNA V3 region amplicon dataset.

## Results and discussion

**Figure 1:**
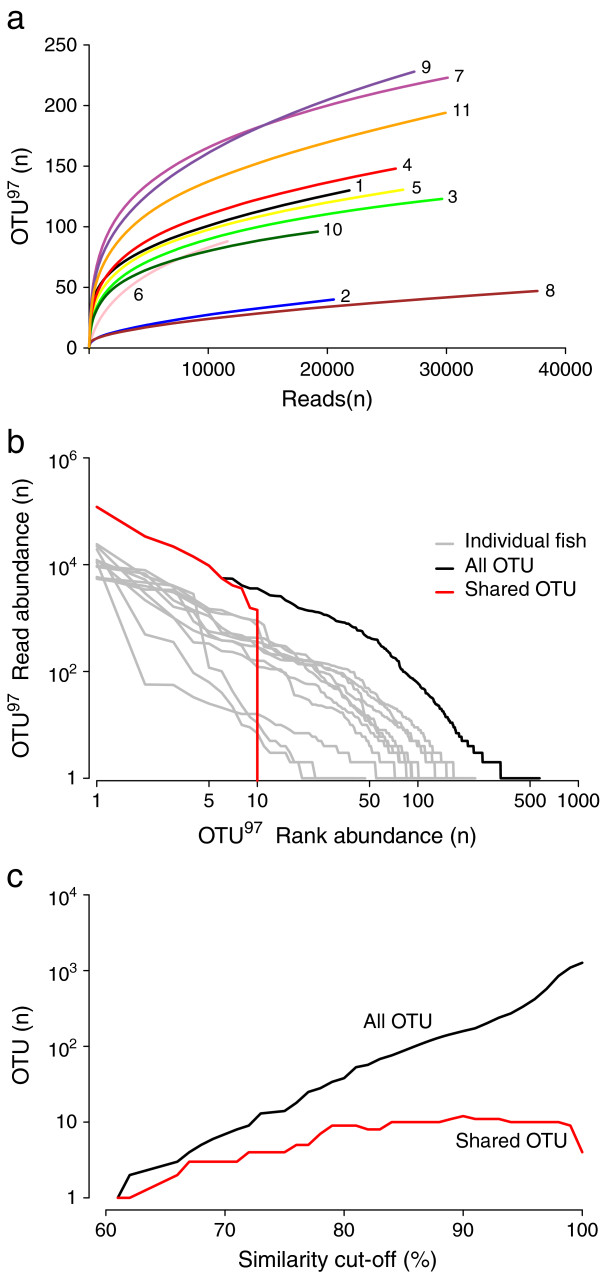
**Wild-caught Atlantic cod have a variable microbial intestinal community. (a)** Rarefaction curve analysis showing the number of detected OTUs per sample based on read number for 11 specimens. Sequences are clustered using a pairwise similarity cut-off of 97%. **(b)** A limited number of highly abundant OTUs (based on read number) are identified in all specimens by comparing rank abundance plots of all OTUs (97% similarity, black) to OTUs that are shared (red). Individual rank abundances (grey) show variation among specimens. **(c)** The total number of detected OTUs (black) and the number of OTUs shared (red) depends on sequencing similarity cut-off values.

We obtained 280447 sequences of approximately 200 basepair (bp) of the 16S rRNA V3 region and identified 573 OTUs at 97% sequence similarity. Rarefaction curve analysis, depicting the relationship between the number of detected OTUs and read number, shows that the final number of OTUs per sample (ranging from 40 to 228) is not caused by uneven sequencing depth (Figure 1a). This variation among samples also appears in estimates of community diversity (based on Shannon and Inverse Simpson indices), which vary an order of magnitude (Table 1). Analogous to the intestinal community composition in zebrafish [17] or human e.g. [9], the samples are typically dominated by a few abundant OTUs, while the majority of OTUs is present at rare frequency (e.g., 62% of the 573 OTUs occur once). At 97% sequence similarity, 10 OTUs are shared that are highly abundant based on the number of reads (Figure 1b). The number of reads assigned to these OTUs varies substantially among individuals, and no more than five OTUs are shared using a detection cut-off value of at least five reads (reflecting a 99% detection probability assuming a binominal distribution, Additional file 1: Table S1). Moreover, for sequence similarity values above 80% the number of shared OTUs is fairly constant, indicating that this number is not a result of restrictive cut-off values when clustering (Figure 1c). Overall, the shared OTUs represent a fraction of the overall sequence diversity for a wide range of cut-off values (Figure 1c).

**Table 1:** Alpha diversity estimates of the Atlantic cod intestinal microbial community

	**OTU**		**Shannon index**		**Inverse Simpsons index**	
**Specimen**	***μ***	***σ***	***μ***	***σ***	***μ***	***σ***
1	97	4.03	2.62	0.01	7.36	0.10
2	26	2.60	0.30	0.01	1.12	0.00
3	89	3.83	1.22	0.02	1.74	0.02
4	108	4.24	2.10	0.02	3.71	0.05
5	96	3.83	2.63	0.01	8.59	0.10
6	73	3.21	0.32	0.01	1.09	0.00
7	163	4.94	2.80	0.02	6.50	0.10
8	24	2.70	1.08	0.01	2.18	0.02
9	158	5.44	3.07	0.01	11.18	0.16
10	77	3.26	1.59	0.02	2.33	0.03
11	136	4.84	2.44	0.02	5.26	0.07

Normalized mean values (*μ*) and standard deviations (*σ*) for the number of OTUs, Shannon index and Inverse Simpsons index. Normalized values were obtained by random resampling according to the smallest sample size (n = 11625, specimen 6) and standard errors were obtained by bootstrapping (n = 1000). OTUs are clustered according to a 97% sequence similarity cut-off value.

**Figure 2:**
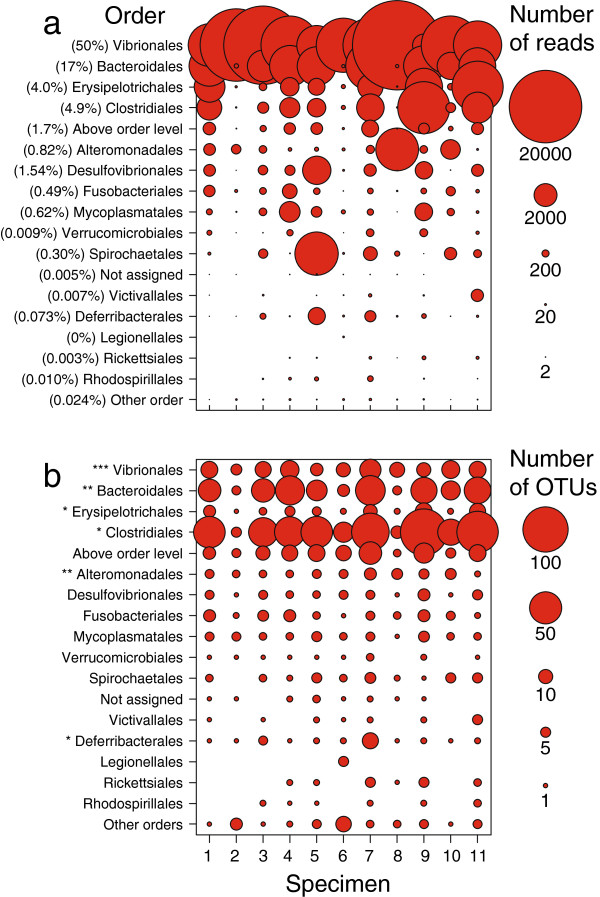
**Taxonomical community composition of the intestinal microbial community in Atlantic cod. (a)** Individual sequence read number per order, illustrated by circle surface area, show a variable microbial community composition. Members from the order Vibrionales are most abundant, followed by those from the orders Bacteriodales, Erysipelotrichales and Clostridiales. The number of reads beloning to particular taxonomic classifications can fluctuate several orders of magnitude among specimens. Overall read number per order for all specimens (% median read number) is given in front of the order name. **(b)** The number of individual OTUs detected per order (97% sequence similarity), illustrated by circle surface area, show that the taxonomically most diverse orders are not necessarily the most abundant based on the number of sequence reads. The presence of a shared OTU (*) is indicated in front of the order name.

At order level classification, the intestinal community of Atlantic cod is dominated by Vibrionales followed by variable numbers of Bacteroidales, Erysipelotrichales and Clostridiales (Figure 2a). The high proportion Vibrionales (50%) agrees with those proportions found in a meta-analysis based on GenBank sequences of other marine carnivores [11] and bacteria from this order have also previously been isolated from the Atlantic cod gut e.g. [18]. Nevertheless, the intestinal community also contains a substantial proportion of Bacteriodales (17%). Such abundance has previously been proposed to be a characteristic of the microbial community of marine herbivores, and this finding suggests that the distinction between herbivorous and carnivorous fish may be more subtle [11]. Members of the most abundant orders agree with those reported previously in Atlantic cod using both culture-dependent and culture-independent techniques [18-22]. In addition, using high throughput sequencing, several more orders are detected that are relatively rare. Shared OTUs belong to the orders Vibrionales, Bacteroidales, Erysipelotrichales, Clostridiales, Alteromonadales and Deferribacterales (Figure 2b). Overall, taxonomical diversity (based on number of OTUs per order) does not necessarily correlate to the number of reads per order.

To our knowledge, our dataset provides the first characterization using high throughput sequencing of individual intestinal microbial community structure in a natural population of marine fish. It is possible that our sampling retrieved fish from different populations. Nevertheless, tagging studies in the Norwegian Skagerrak coastal region have shown that adult Atlantic cod have confined home ranges [23] and genetic studies have revealed fine scale geographical population structure [24,25]. Considering our single sample location, far from the fjord exit, and comparable size of individuals (Additional file 1: Table S2), we assume that our individuals were retrieved from a local population experiencing similar environmental conditions.

Among our samples, we find 10 shared OTUs, with profound variation in the number of reads per individual. This number of shared OTUs may be an overestimation as the fish were kept in a single tank after capture and thus experienced the same environmental conditions before sampling. We presume such a similar environment is more likely to homogenize microbial communities, rather than promote individual differences. Nevertheless, this shared number of OTUs appears relatively low compared to the number of shared OTUs (21 OTUs, at 97% sequence identity cut-off) among populations of zebrafish from radically different environmental conditions, coming either from natural populations in India or from artificial environments in two separate laboratories in the USA [17]. For now, this difference in shared OTUs between our study and the study focusing on zebrafish is difficult to interpret due to methodological variation e.g. pooled versus individual samples, V1-V2 versus V3 16S rRNA region, [17]. It will be interesting to investigate if these differences in shared OTUs membership are environmentally determined (e.g., a largely different food preference and habitat) or are species specific (e.g., the unusual Atlantic cod immune system which might affect its host-microbe interactions [12,13]).

Community diversity estimates based on 454 amplicon data are influenced by methodological factors such as fragment length, PCR bias and choice of 16S rRNA gene region. Specifically, shorter amplicon lengths (e.g. < 400 bp) may result in relatively higher diversity estimates compared to longer fragments [26] and arguably provide a better assessment of community structure [27]. In contrast, species richness estimate based on analyses of the 16 s rRNA V3 region appears to slightly underestimate diversity relative to the full-length gene [28]. Such methodological issues make it difficult to compare community diversity across different studies [29], although metrics that use both richness and relative abundance (i.e. Shannon and Inverse Simpson indices) appear robust [30], in particular considering our extensive sequencing depth [31]. Interestingly, these metrics fluctuate several orders of magnitude among our different specimens, and show large individual variation in community composition and diversity. The most diverse individuals appear to have a comparable community complexity relative to those found in humans [7,32].

A variety of properties, such as shared OTU membership, shared phylogeny, persistence or connectivity can be used to define microbial cores [33]. Here we investigated a core microbiota based on shared membership. Definitions for such a core have been proposed ranging from a lineage present in more than half the population [3] to an abundant lineage shared among all individuals [8]. We argue that the utility of such concept depends on the specificity with which it describes a biological phenomenon and favor the idea that a lineage should be reliably identified among all individuals in order to belong to a core microbiota, hence with a detection probability of at least 99%. According to such definition, the presence of a core microbiota at 97% sequence similarity consists of five OTUs in this population of Atlantic cod. These OTUs belong to orders Vibrionales, Bacteroidales, Erysipelotrichales, Clostridiales and Alteromonadales.

It is possible that the observation of a shared OTU membership can be explained by other factors other than host-specific selection. For example, between teleost fish, the colonization and community structure of the microbial gut community appears better explained by environmental factors such as food choice or habitat (i.e. salinity) than by host phylogeny [11,34]. Considering our single sample location, it is currently unclear if the observed core microbiota in Atlantic cod is explained by host-specific selection or driven by shared environmental factors. Interestingly, human microbial gut communities are functionally remarkably similar, despite extensive variation in taxonomic composition [7-9]. This functional redundancy may provide support for a ‘founder takes all’ process of colonization, in which a successful colonizer can prevent the subsequent colonization by other, functionally similar strains through high density blocking [35]. Such a stochastic process could lead to the high variation in community composition that we observe among our different specimens.

## Conclusions

Based on the extensive 454 sequencing of a 16S rRNA V3 region amplicon library, we find that the OTU based community diversity estimates of the intestinal microbial community in wild-caught Atlantic cod vary significantly among individuals collected at a single location. This individual level variation suggests that a complex combination of factors influences the microbial species distribution in these intestinal communities. Importantly, such variation has gone unobserved in previous studies of natural populations of teleosts whereby samples of pooled individuals were analyzed [11,17], which may affect estimates of the number of shared OTUs among hosts.

## Methods

Live Atlantic cod were collected at a single location (N59.871278, W10.587208) using a fish trap in the Oslo fjord, Norway (Additional file 1) and transported to an animal facility approved by the Norwegian Animal Research Authority (NARA, http://oslovet.norecopa.no/dokument.aspx?dokument=67, approval number 155/2008). The specimens were kept in a common tank (2000 l), at ambient water temperature and light conditions (i.e., 6°C and L:D 8:16, respectively) without feed for between seven and twelve days before sampling to help reduce variation in community composition due to the presence of food items [11]. The fish were humanely sacrificed by a blow to the head (without any administration of other sedatives) before sampling. The experiments were approved by NARA’s authorized representative at the facility and were conducted in accordance with the European Convention for the protection of vertebrate animals (http://conventions.coe.int/treaty/en/treaties/html/123.htm) used for experimental and other scientific purposes.

Flushed contents from the intestinal tract, including the rectal portion and excluding the stomach, were used for DNA isolation (Additional file 1) and PCR amplification after which individually barcoded amplicons were pooled and sequenced using 454 technology [36]. Sequence data was binned into the individual samples using the barcoded tags and subsequently cleaned from artifacts. Diversity estimates were calculated using Mothur [37]. Representative OTU sequences were compared to the SILVA SSU ref NR V108 database [38] (http://www.arb-silva.de) using BlastN [39] and classified using the LCA algorithm in Megan [40].

## Competing interests

The author declare that they have no competing interests.

## Authors’ contributions

BS and SJ conceived and designed the experiments. TH, BS and SJ performed the experiments. TH extracted DNA and created the amplicon libraries. BS and TH analyzed the data. BS, TH, SJ, and KJ wrote the manuscript. All authors read and approved the final manuscript.

## Supplementary Material

Additional file 1 Supplementary section containing detailed methods, analyses and supplementary tables. Click here for file
