# Supplementary material for: Next generation sequencing shows high variation of the intestinal microbial species composition in Atlantic cod caught at a single location
Source: BMC Microbiol. 2013 Nov 9;13:248. doi: 10.1186/1471-2180-13-248 (PMC3840566; doi:10.1186/1471-2180-13-248)
Supplement: Additional file 1 — Supplementary section containing detailed methods, analyses and supplementary tables. [file 1471-2180-13-248-S1.pdf]

Additional file

Next generation sequencing shows high variation of the intestinal microbial species composition in Atlantic cod caught at a single location

Bastiaan Star, Thomas H.A. Haverkamp, Sissel Jentoft & Kjetill S. Jakobsen

Contents:

Sampling ... 1
Sample preparation and 454 sequencing ... 2
Quality control ... 4
Data analysis ... 5

Sampling

Live Atlantic cod specimens (*Gadus morhua*) were obtained from the inner Oslo fjord from one location (N59.871278, W10.587208) between 28-10-2011 and 03-11-2011 and were transported to the laboratory facilities at Blindern, University of Oslo (UiO), Norway, which is an animal facility approved by the Norwegian Animal Research Authority (NARA, http://oslovet.norecopa.no/dokument.aspx?dokument=67, approval number 155/2008). Fish were kept in a single tank (2000 l) without feed or further intervention for a minimum of seven days and a maximum of twelve days before sampling. Temperature and light regime were ~ 6°C and L:D 8:16, respectively and welfare of fish was inspected on a daily basis. The handling of specimens and experiments were approved by NARA's authoritative representative ("ansvarshavende") at the facility and were conducted in accordance with the European Convention for the protection of vertebrate animals (http://conventions.coe.int/treaty/en/treaties/html/123.htm) used for experimental and other scientific purposes. All specimens were handled separately for dissection. For each specimen, we recorded length, weight, gonad weight, sex, and the presence of parasites in the gut (Supplementary Table 2). Between samples, gloves were changed and equipment was cleaned with ethanol to minimize the risk of cross-contamination of intestinal samples. Whole intestines, excluding stomach and including the rectal section were dissected and flushed with rRNAlater (Life Technologies Ltd, UK). The RNA later cell suspension was collected and stored at 4°C until DNA isolation.

Sample preparation and 454 sequencing

DNA isolation was initiated by mixing the RNA later cell suspension mix with Phosphate Buffered Saline (Life Technologies Ltd, UK). The suspension was centrifuged for 10 minutes at 4°C 4800 g using a Hereaus Multifuge X3R Centrifuge (Thermo Scientific, USA). Supernatant was removed and the pellet was split in three fractions. Each fraction was transferred to a 2 ml tube filled with beads (FastDNA spin kit for soil (MPbiomedicals, France). An additional blank sample was included to monitor contamination during DNA extraction. The DNA extraction protocol provided with the FastDNA spin kit for soil was used with the following modification. Bead beating was performed for 30 seconds at speed 6.0 with a MP FastPrep-24 bead beater (MPbiomedicals, France). After bead beating the protocol was followed according to the manufacturer's instructions and the triplicate DNA extracts from each fish were pooled. In order to prevent PCR inhibition, DNA extracts were cleaned using the PowerClean DNA Clean-Upkit (Mobio Laboratories, Carlsbad, CA, USA) following the manufacturer's instructions. DNA concentration was measured using a Nanodrop ND1000 (Nanodrop Technologies, Wilmington, DE, USA).

For each individual sample, we set up 25 µl PCR reactions in triplicate to amplify the V3 regions with the following contents: 10 ng of template per reaction; 5uM of each barcoded PCR primer 338F and 533R [1] (Supplementary Table 3); 10mM dNTPs , 1X HF PCR buffer (Thermo Scientific, Waltham, MA, USA), 0.02 units/ µl Phusion Hot Start II DNA Polymerase. The PCR program was run on a Biometra T1 Thermocycler with the following program: a hotstart for 60 seconds at 98°C, followed by 20 cycles of 10 seconds at 98°C, 30 seconds at 55°C and 15

seconds at 72°C. The reaction finished with a 10 minute elongation step at 72 °C. The correct size of the PCR products was confirmed by visual inspection of a 2% agarose gel (Seakem LE agarose, Lonza group ltd, Basel, Switzerland).

To normalize the PCR sample input for pyrosequencing we used the SequalPrep Normalization Plate (96) kit (Invitrogen, Paisley, UK) and followed the instructions provided by the manufacturer. 15 µl of each PCR reactions were bound to the normalization plates. Elution was done with 20 µl elution buffer, and the eluent of each normalization was combined in one 2 ml eppendorf tube. The amplicon library was concentrated using the SV gel / PCR clean-up system(Promega, Fitchburg, WI, USA) following the manufacturer's instructions. The sample was eluted in 50 µl water twice to collect as much PCR product as possible. The concentrated products were loaded on a 2% agarose gel to separate the PCR product from the primer dimers. The PCR product was excised from the agarose gel using a sterile razor, and transferred to a clean eppendorf tube for gel extraction with the SV gel / PCR clean-up system (Promega, Fitchburg, WI, USA). Gel extraction was performed following the manufacturers' instructions. The sample was eluted in 50 µl water. After gel extraction the amplicon library was checked for residual primer dimers by loading 4 µl onto a 2 % agarose gel. Sequencing of the amplicon library was executed using Lib-L chemistry on half a plate with a 454 GS-FLX pyrosequencer [2] at the Norwegian Sequencing Centre (UiO, Oslo).

Quality control

After sequencing, sequence data and associated quality scores were extracted from the sff file using Mothur [3]. The Schloss SOP was followed for the quality control. In brief, the sequences were separated into the different samples using the forward primers and individually barcoded tags. The pyronoise implementation shhh.flows in Mothur was used to reduce sequence noise. Subsequently, sequences containing tags with mismatches were discarded, while simultaneously removing these tags and forward primers from the remaining sequences. The reverse primers and tags were removed by aligning the sequences against the SILVA SSU ref NR V108 database and sequences that could not be aligned were discarded. Manual inspection indicated that the majority of these sequences was closely related to *Gadus morhua* mitochondrial sequences. The sequences were preclustered, binning sequences with a difference of two or less SNPs [4]. Finally, chimeric sequences were identified and removed using the chimera.uchime option in Mothur, resulting in a dataset of 280477 reads, which was used in the subsequent diversity analyses.

Data analysis

The obtained dataset was used for generating Operational Taxonomical Units (OTUs) through average neighbor clustering based on pairwise distances between the sequences using Mothur. Rarefaction curves were calculated using a 97% similarity cut-off. The OTU Rank abundance curve was created using the abundance of reads per OTU for each sample. Figures were plotted in R (v 2.15.0) and finalized for publication using Adobe Illustrator. The number of OTUs, Shannon index and inverse Simpson index were calculated based on datasets that were normalized towards the smallest sample size (n=11625). Confidence intervals were calculated by bootstrapping (n=1000) using Matlab.

Representative OTU sequences were extracted from each sample. The sequences were compared against the SILVA SSU ref NR V108 database using BlastN. The Blast results were imported into MEGAN and the representative OTU sequences were classified using the standard Lowest Common Ancestor parameters except: Minscore: 100, Minsupport: 1, TopPercent: 1 [5]. To generate Figure 2a we used the number of reads assigned to each representative OTU sequence to calculate the number of reads assigned to each order classification per sample.

Supplementary Table 1

Number of sequence reads per sample for OTUs shared among all specimens. OTUs are clustered according to a 97% sequence similarity cut-off value. OTUs that are shared with a minimum number of five reads per specimen are indicated with bold text.

OTU ID	Classification (order)	Specimen										
		1	2	3	4	5	6	7	8	9	10	11
**0001**	Vibrionales	2058	19404	22255	12080	179	11137	9110	24263	176	12314	7879
**0005**	Bacteroidales	5879	63	2918	5232	1407	21	6805	43	5430	2159	3836
**0006**	Erysipelotrichales	3424	22	232	1312	947	57	2157	7	3978	168	9528
**0007**	Vibrionales	3376	494	1033	320	5678	25	662	23	1342	303	872
0008	Vibrionales	1239	10	59	55	1	8	1409	6123	307	365	9
**0018**	Alteromonadales	157	9	122	18	6	33	128	4049	5	1013	18
0063	Bacteroidales	4	7	2	8	4047	10	13	1	5	1	1
0024	Alteromonadales	132	356	111	31	10	4	26	2830	1	94	1
0011	Clostridiales	393	3	26	160	15	5	319	1	109	17	504
0105	Deferribacterales	1	1	119	6	1158	16	1	14	92	2	8

Supplementary Table 2

Metadata associated with 11 Atlantic cod specimens caught in the Oslo Fjord.

Specimen	Size (cm)	Weight (kg)	Gonad weight (gr)	Gender (m/f)	Parasites (y/n)
1	57	1.35	28.8	m	n
2	49	1.12	36.7	f	n
3	50	1.05	39.2	m	n
4	59	1.77	34.6	m	n
5	63	2.6	52.4	f	n
6	54	1.32	11.6	m	n
7	54.5	1.42	39.5	m	n
8	52	1.08	9	m	n
9	61	1.82	33.9	m	n
10	62	1.72	125	m	y
11	55	1.47	6.2	f	n

Supplementary Table 3

16S rRNA – V3 region PCR primers with MID index tag for each individual fish.

Sample	Primer name	Adaptor sequence	MID -tag	Primer sequence
		Forward primer (A lib-L chemistry)		
FISH01	TAG01_F338	CCATCTCATCCCTGCGTGTCTCCGACTCAG	acgac	ACTCCTACGGGAGGCAGCAG
FISH02	TAG02_F338	CCATCTCATCCCTGCGTGTCTCCGACTCAG	actgt	ACTCCTACGGGAGGCAGCAG
FISH03	TAG03_F338	CCATCTCATCCCTGCGTGTCTCCGACTCAG	agagc	ACTCCTACGGGAGGCAGCAG
FISH04	TAG04_F338	CCATCTCATCCCTGCGTGTCTCCGACTCAG	atatg	ACTCCTACGGGAGGCAGCAG
FISH05	TAG05_F338	CCATCTCATCCCTGCGTGTCTCCGACTCAG	tactg	ACTCCTACGGGAGGCAGCAG
FISH06	TAG06_F338	CCATCTCATCCCTGCGTGTCTCCGACTCAG	tatgc	ACTCCTACGGGAGGCAGCAG
FISH07	TAG07_F338	CCATCTCATCCCTGCGTGTCTCCGACTCAG	cgacg	ACTCCTACGGGAGGCAGCAG
FISH08	TAG08_F338	CCATCTCATCCCTGCGTGTCTCCGACTCAG	ctgtc	ACTCCTACGGGAGGCAGCAG
FISH09	TAG09_F338	CCATCTCATCCCTGCGTGTCTCCGACTCAG	cgtat	ACTCCTACGGGAGGCAGCAG
FISH10	TAG10_F338	CCATCTCATCCCTGCGTGTCTCCGACTCAG	attct	ACTCCTACGGGAGGCAGCAG
FISH11	TAG11_F338	CCATCTCATCCCTGCGTGTCTCCGACTCAG	cacac	ACTCCTACGGGAGGCAGCAG
Blank	Blank_F338	CCATCTCATCCCTGCGTGTCTCCGACTCAG	agcag	ACTCCTACGGGAGGCAGCAG
		Reverse primer (B lib-L chemistry)		
FISH01	TAG01_R533	CCTATCCCCTGTGTGCCTTGGCAGTCTCAG	acaac	TTACCGCGGCTGCTGGCA
FISH02	TAG02_R533	CCTATCCCCTGTGTGCCTTGGCAGTCTCAG	tctaa	TTACCGCGGCTGCTGGCA
FISH03	TAG03_R533	CCTATCCCCTGTGTGCCTTGGCAGTCTCAG	caatc	TTACCGCGGCTGCTGGCA
FISH04	TAG04_R533	CCTATCCCCTGTGTGCCTTGGCAGTCTCAG	acgta	TTACCGCGGCTGCTGGCA
FISH05	TAG05_R533	CCTATCCCCTGTGTGCCTTGGCAGTCTCAG	taacg	TTACCGCGGCTGCTGGCA
FISH06	TAG06_R533	CCTATCCCCTGTGTGCCTTGGCAGTCTCAG	agaca	TTACCGCGGCTGCTGGCA
FISH07	TAG07_R533	CCTATCCCCTGTGTGCCTTGGCAGTCTCAG	catca	TTACCGCGGCTGCTGGCA
FISH08	TAG08_R533	CCTATCCCCTGTGTGCCTTGGCAGTCTCAG	tacgc	TTACCGCGGCTGCTGGCA
FISH09	TAG09_R533	CCTATCCCCTGTGTGCCTTGGCAGTCTCAG	agctc	TTACCGCGGCTGCTGGCA
FISH10	TAG10_R533	CCTATCCCCTGTGTGCCTTGGCAGTCTCAG	aggag	TTACCGCGGCTGCTGGCA
FISH11	TAG11_R533	CCTATCCCCTGTGTGCCTTGGCAGTCTCAG	ctagt	TTACCGCGGCTGCTGGCA
Blank	Blank_R533	CCTATCCCCTGTGTGCCTTGGCAGTCTCAG	cttag	TTACCGCGGCTGCTGGCA

Supplementary References

1. Huse SM, Dethlefsen L, Huber JA, Welch DM, Relman DA, Sogin ML: **Exploring Microbial Diversity and Taxonomy Using SSU rRNA Hypervariable Tag Sequencing**. *PLoS Genet* 2008, **4**(11):e1000255.
2. Margulies M, Egholm M, Altman WE, Attiya S, Bader JS, Bemben LA, Berka J, Braverman MS, Chen YJ, Chen ZT *et al*: **Genome sequencing in microfabricated high-density picolitre reactors**. *Nature* 2005, **437**(7057):376-380.
3. Schloss PD, Westcott SL, Ryabin T, Hall JR, Hartmann M, Hollister EB, Lesniewski RA, Oakley BB, Parks DH, Robinson CJ *et al*: **Introducing mothur: Open-Source, Platform-Independent, Community-Supported Software for Describing and Comparing Microbial Communities**. *Applied and Environmental Microbiology* 2009, **75**(23):7537-7541.
4. Huse SM, Welch DM, Morrison HG, Sogin ML: **Ironing out the wrinkles in the rare biosphere through improved OTU clustering**. *Environmental Microbiology* 2010, **12**(7):1889-1898.
5. Huson D, Richter D, Mitra S, Auch A, Schuster S: **Methods for comparative metagenomics**. *BMC Bioinformatics* 2009, **10**(Suppl 1):S12.
